# Decoupling of DNA methylation and activity of intergenic LINE-1 promoters in colorectal cancer

**DOI:** 10.1080/15592294.2017.1300729

**Published:** 2017-03-16

**Authors:** Natasha Vafadar-Isfahani, Christina Parr, Lara E. McMillan, Juliane Sanner, Zhao Yeo, Stephen Saddington, Oliver Peacock, Hazel A. Cruickshanks, Richard R. Meehan, Jonathan N. Lund, Cristina Tufarelli

**Affiliations:** a School of Medicine, Royal Derby Hospital, University of Nottingham, Derby, UK; b School of Biological Sciences, University of Edinburgh, Edinburgh, UK; c MRC Human Genetics Unit, Institute of Genetics and Molecular Medicine, University of Edinburgh, Edinburgh, UK

**Keywords:** CRC, chromatin, colorectal cancer, H4K20me3, L1PA2, LINE-1, LCT, L1-ASP, methylation, retrotransposons

## Abstract

Hypomethylation of LINE-1 repeats in cancer has been proposed as the main mechanism behind their activation; this assumption, however, was based on findings from early studies that were biased toward young and transpositionally active elements. Here, we investigate the relationship between methylation of 2 intergenic, transpositionally inactive LINE-1 elements and expression of the LINE-1 chimeric transcript (LCT) 13 and LCT14 driven by their antisense promoters (L1-ASP). Our data from DNA modification, expression, and 5′RACE analyses suggest that colorectal cancer methylation in the regions analyzed is not always associated with LCT repression. Consistent with this, in HCT116 colorectal cancer cells lacking DNA methyltransferases DNMT1 or DNMT3B, LCT13 expression decreases, while cells lacking both DNMTs or treated with the DNMT inhibitor 5-azacytidine (5-aza) show no change in LCT13 expression. Interestingly, levels of the H4K20me3 histone modification are inversely associated with LCT13 and LCT14 expression. Moreover, at these LINE-1s, H4K20me3 levels rather than DNA methylation seem to be good predictor of their sensitivity to 5-aza treatment. Therefore, by studying individual LINE-1 promoters we have shown that in some cases these promoters can be active without losing methylation; in addition, we provide evidence that other factors (e.g., H4K20me3 levels) play prominent roles in their regulation.

## Abbreviations

LINE-1: long interspersed element 1
LCT: LINE-1 chimeric transcript
L1-ASP: LINE-1 antisense promoter
5-aza: 5-azacytidine
hMeDIP: hydroxymethylated DNA immunoprecipitation
5hmC: 5-hydroxymethylcytosine
ChIP: chromatin immunoprecipitation

## Introduction

Long interspersed element 1 (LINE-1) regions are repetitive DNA sequences that comprise about 17% of the human genome, equivalent to approximately 8 times the protein coding portion of the genome.^1^ LINE-1s are autonomous mobile elements that carry their own promoters and the information to copy and paste themselves to different locations in the genome, a phenomenon known as retrotransposition.^2^ Of the about 516,000 LINE-1s present in the human genome, just around 7000 have retained potentially intact promoters; of these, nearly 5000 are full-length but only up to 20 so called ‘hot-L1s’ have been shown to be able to retrotranspose.^3–5^

It is now becoming more widely accepted that LINE-1s can also contribute to tumor in ways that are not related to their mobilization.^6–8^ Some of these effects (e.g., the ability of LINE-1 promoters to act as alternative promoters for protein coding genes^9^) are due to transcription from the LINE-1 promoters and therefore can be exerted by all elements carrying intact promoters and not restricted to the few hot-L1s. LINE-1s contain an internal bidirectional promoter within their 5′ untranslated region (L1–5′UTR): a sense promoter (L1-SP) responsible for transcription of the element itself, and an antisense promoter (L1-ASP) driving transcription away from the element.^10,11^ L1-ASP activity has been mapped between positions 400 and 600 of the L1–5′UTR using transgenic constructs carrying retrotransposition competent LINE-1s.^12^ L1-ASP activity in this region was further confirmed in human embryonic stem and carcinoma cells.^13^ More recently, this activity has been shown to drive expression of the ORF0 protein with transposition enhancing properties and coded by about 781 LINE-1 loci in the human genome.^14^ Transcription initiation from L1-ASP in cancer has also been found between position 160 and 200 of the L1–5′UTR, in particular for intergenic, retrotransposition deficient LINE-1s.^10,15^

It has been proposed that DNA methylation has evolved to prevent potentially damaging effects by silencing endogenous retrotransposons.^16,17^ Indeed, in somatic cells these elements are silenced and heavily methylated.^18-20^ By contrast, hypomethylation of LINE-1s is a common feature of cancer cells,^21^ which has been associated with LINE-1s activation and consequent further promotion of genome instability and cancer progression.^22–24^ In general, LINE-1 methylation levels decrease as the severity index of the cancer increases.^25–27^ LINE-1 activity and new somatic LINE-1 insertions have been described in several cancers of epithelial cell origin and happen at particularly high frequencies in colorectal tumors.^28^ These observations have led to the notion that hypomethylation is the primary cause of activation of LINE-1 promoters in cancer. However, the evidence supporting this order of events is not conclusive.

Given their abundance and propensity to be methylated, LINE-1 methylation levels have been often used as a surrogate measure for global DNA methylation.^29,30^ However, until recently, the majority of LINE-1 methylation studies have looked at global levels using methods that favor detection of young and transpositionally active elements.^31^ These global studies lack resolution and assume one general regulatory network or machinery for all LINE-1 elements within the genome irrespective of cellular context. This is an unlikely scenario since LINE-1 elements can be found in a variety of loci and carry individual or familial SNPs. In agreement with this, there are numerous individual examples of LINE-1 elements that do not conform to the global methylation trends. Smith et al.^32^ identified a subset of LINE-1 elements that were hypomethylated in oocytes despite the majority of transcription elements maintaining a heavy methylation status. In cancer, Phokaew et al.^33^ showed that some LINE-1s are not affected by global hypomethylation. Similarly, generalized activation of LINE expression was not observed in mouse hypomethylation models.^34,35^

These observations highlight the importance of studying individual LINE-1 elements to advance our understanding of their regulatory mechanism. Despite this, locus-specific LINE-1 studies are sparse and they are mainly focused on elements located within the body of annotated genes (intragenic LINE-1s) and their immediate effect on their host gene.^36,37^ We previously identified transcripts initiating at L1-ASP, which we referred to as LINE-1 chimeric transcripts or LCTs because they contain within the same molecule both LINE-1 and unique sequences.^15^ Of particular interest, 2 LCTs (LCT13 and LCT14) were found to initiate at the antisense promoter (L1-ASP) of transpositionally inactive L1PA2 elements located at intergenic regions (between genes) on human chromosomes 7 and 5 respectively.^15,38^ We further demonstrated that in up to 50% of colorectal cancers lacking expression of the tumor suppressor gene *TFPI-2*, this silencing is associated with the presence of the overlapping anti-sense LCT13 transcript, suggesting that its expression may have a functional consequence in cancer.^38^ In an ES cell line model an antisense transcript can silence *TFPI-*2 expression before de novo methylation of its promoter in differentiated cells.^38^ Our initial observations in MCF10A non-neoplastic breast cells suggested that in breast hypomethylation is involved in activation of the promoters of these intergenic L1PA2s^15^; however, it remains unclear whether loss of DNA methylation is always necessary to activate them in cancer. In this study, we have investigated at a locus-specific level if loss of DNA methylation within the L1–5′UTR of the LINE-1s driving LCT13 and LCT14 is always associated with their expression, and we have also compared the presence of histone marks associated with active and inactive chromatin states to transcriptional activity and DNA methylation levels at these sites.

## Results

### Relationship between activity and methylation of LCT13 in cancer

**Figure 1.**
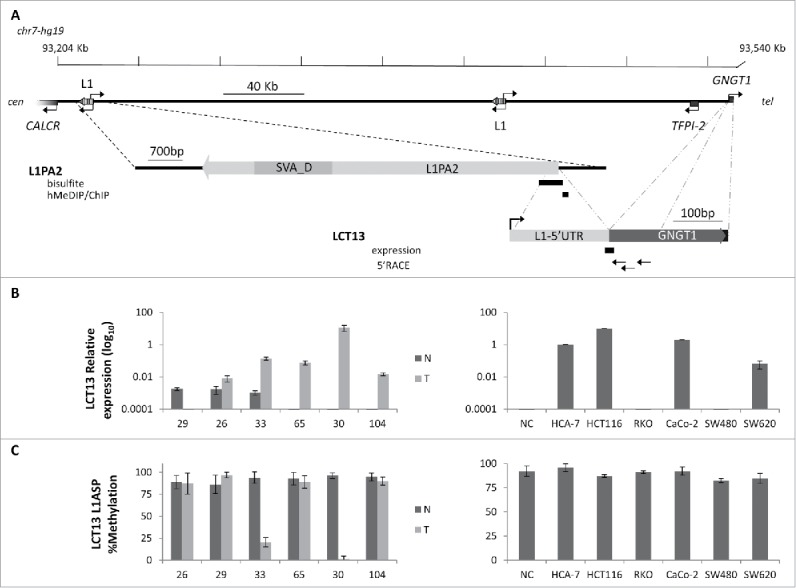
Relationship between methylation and expression of LCT13 L1ASP in CRC. (A) Top: Schematic diagram of the LCT13 genomic locus on human chromosome 7 (chr7:93,204,042–93,540,485; center) with indicated the positions of the *CALCR, TFPI-2*, and *GNGT1* genes and of the 2 intact intergenic LINE1s (L1) present in this region. Middle: enlargement of the LINE-1 (L1PA2: chr7:93,213,393–93,221,079, with an SVA_D spanning the interval 93,214,544–93,216,214) from which LCT13 originates with the regions (black bars) tested by bisulfite or hMeDIP and ChIP. Bottom: enlargement of the LCT13 spliced transcript with indicated its exon structure [LINE-1 5′UTR fragment in light gray (chr7:93,220,882–93,221,083) and, in dark gray, the 2 GNGT1 exons (93,536,051–93,536,154 and 93,540,102–93,540,235), part of the LCT13 transcript]. Also indicated are the positions of the Taqman assay used for LCT13 expression studies located at the splice junction (black bar) and of the primers used for 5′RACE (arrows). All coordinates are from hg19 annotations; scale is in kilobase pairs (kb). (B) Bar charts showing the expression of LCT13 measured by real time RT-PCR and expressed relatively to the geometric mean of 3 reference genes in matched normal (dark gray, N) and tumor (light gray, T) tissues from 6 colorectal cancer patients (left panel) and 6 cell lines (right panel). NC: normal colon, commercially sourced total RNA from 7 healthy donors pooled together. (C) Bar charts of the methylation levels measured by bisulfite sequencing in the tissues of the 6 patients and cell lines presented in B.

We previously showed that aberrant activation of LINE-1 antisense promoters in cancer can occur at older and transposition deficient elements,^15^ such as the L1-ASP of the L1PA2 that drives expression of LCT13 (Fig. 1A).^38^ To determine whether this activity was associated with a decrease in methylation, we compared expression of LCT13 to its methylation in matched normal and tumor tissues from 6 colorectal cancer (CRC) donors (26, 29, 30, 33, 65 and 104). In addition, 3 microsatellite instable (MSI: HCA-7, HCT116, RKO) and 3 microsatellite stable (MSS: CaCo-2; SW480 and SW620) CRC cell lines were tested as higher levels of methylation at LINE-1s have been reported in MSI relatively to MSS lines.^36^ The patients used for this study were chosen based on their LCT13 expression profile: 29 expresses LCT13 in the normal mucosa (N) but not in the tumor (T); 26 and 33 express LCT13 in N and at increased levels in T; 30, 65 and 104 expressed LCT13 only in T, the most commonly found pattern (Fig. 1B, left panel).^38^ Using the same quantitative RT-PCR approach in the CRC cell lines, we found that HCT116 express the highest levels of LCT13, followed by CaCo-2, HCA-7 and SW620; by contrast, LCT13 expression in RKO and SW480 is not detectable (Fig. 1B, right panel). We then studied, by PCR on bisulfite treated DNA, the methylation of a region of the L1–5′UTR of the LINE-1 driving expression of LCT13 (bisulfite, Fig. 1A middle diagram) in the same patient tissues and cell lines used for the expression analysis. With the exception of patients 33 and 30, where there was a decrease in methylation in the tumor tissue (T) samples, LCT13 is surprisingly highly methylated (>80%) in both normal (N) and T samples from all other patients studied (Fig. 1C, left panel; Fig. S2A). Moreover, this L1–5′UTR region is also highly methylated in the 6 CRC cell lines, regardless of their expression profile (Fig. 1C, right panel; Fig. S2A). Interestingly, in breast cancer (BC) cell lines the expected relationship between levels of methylation and expression of LCT13 was observed, with increasing amounts of expression corresponding to decreasing levels of methylation. However, while T47D are unmethylated, MCF7 and HCC1954 retain >55% methylation (Fig. S2B and C). These findings suggest that, at least in CRC, loss of methylation may not be necessary for LINE-1 promoter activation, or, alternatively, that LCT13 expression in methylated tissues and cell lines may initiate at a different promoter.

**Figure 2.**
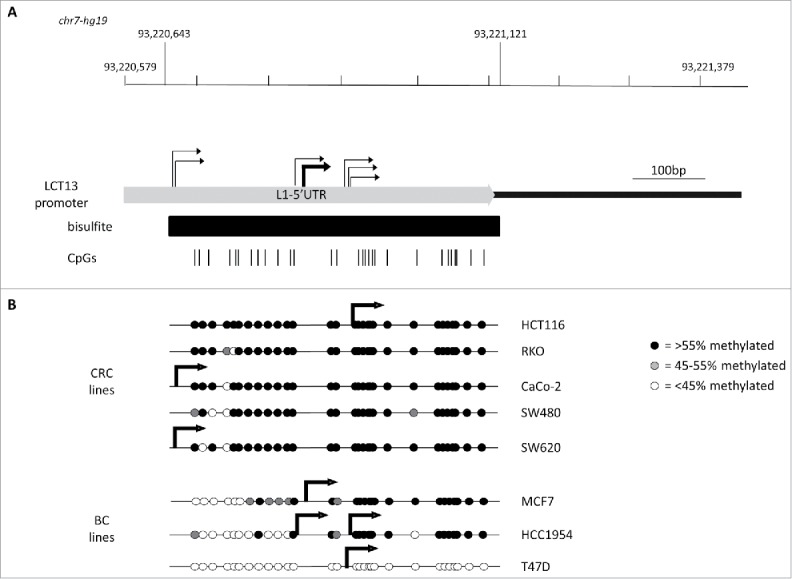
Relationship between L1–5′UTR methylation and LCT13 transcription start sites in cell lines. (A) Schematic diagram showing the 5′UTR of the L1PA2 driving LCT13 (chr7: 93,220,579–93,221,079) with indicated the regions analyzed by bisulfite sequencing (black bar; chr7: 93,220,643–93,221,121) and the positions of the 29 CpG sites (vertical black lines) within it. All coordinates are from hg19 annotations and the scale is in base pairs (bp). Indicated are all the transcription start sites (TSS; bent arrows) identified in the cell lines by 5′RACE demonstrating scattered transcription initiation (light gray) (see also Fig. S3). (B) Diagrams combining the lollipops summarizing the average methylation at each of the 29 CpG site analyzed in the panels of colorectal (CRC) and breast (BC) cancer cell lines (see Fig. S2) with the stronger TSS site identified for the particular cell line (thick bent arrows). No TSS was identified by 5′RACE in RKO and SW480 cell lines, consistent with lack of detectable LCT13 transcripts in these cells (Fig. 1B, right panel).

### LCT13 transcription is driven by L1-ASP

To address whether LCT13 transcription was driven by L1-ASP in the expressing cell lines, we performed 5′RACE anchored in the second exon of the LCT13 transcript (Fig. 1A, bottom diagram). We first optimized conditions in MCF7 cells as we have previously been able to identify LCT13 transcription start sites (TSS) in these cells.^15^ The use of a variety of conditions highlighted the presence of weak and strong TSSs all within L1–5′UTR (Fig. S3). Using conditions optimal for the major TSS, we obtained 5′RACE products for all cell lines analyzed with the exception of RKO and SW480, consistent with the lack of expression of LCT13 in these 2 lines. In all positive cell lines LCT13 transcripts have a major transcription start site (TSS) within L1–5′UTR, confirming that L1-ASP drives LCT13 expression in these cells (Fig. 2A). We next analyzed each TSS in the context of the average methylation of L1–5′UTR within the respective cell line. This revealed that the major TSS was situated within the methylated region of L1–5′UTR in HCT116 and MCF7, while in CaCo-2 and SW620 it lies just outside the CpG rich region studied by bisulfite sequencing (Fig. 2B). In addition, HCC1954 has 2 TSSs that, judging by the relative amounts of 5′RACE products, seem to be used at similar levels: one TSS is in a similar location to that of MCF7 cells; the other is close to the TSS identified in HCT116 and T47D cells and situated within a methylated region (Fig. 2B). This data suggest that, similar to many other RNA polymerase II promoters, the L1-ASP driving LCT13 has scattered TSSs and that in some cases (e.g., HCT116) transcription initiation may occur despite the presence of DNA methylation; alternatively, the detection of methylation by bisulfite may indicate the presence of hydroxymethylated cytosine.

**Figure 3.**
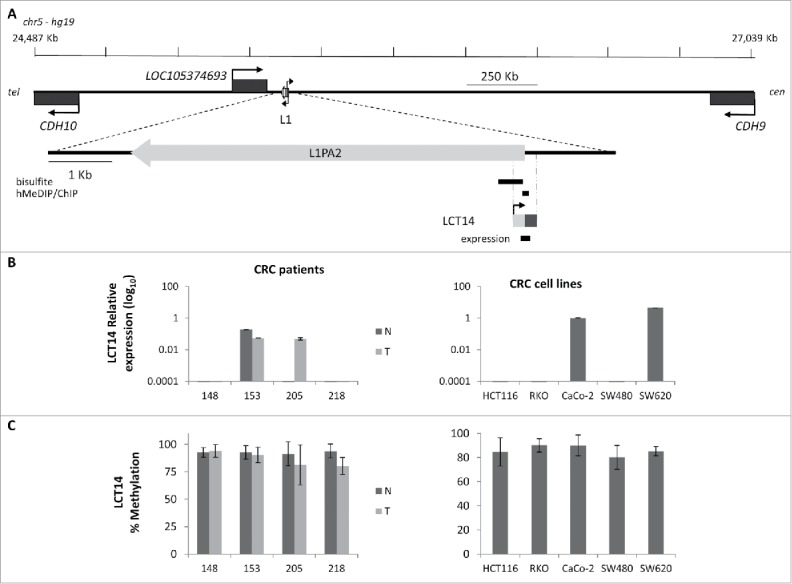
Relationship between methylation and expression of LCT14 in CRC. (A) Schematic diagram of the LCT14 genomic locus on human chromosome 5 (coordinates: 24,487,209–27,038,689) with indicated positions of the annotated genes *CDH10, LOC105374693*, and *CDH9* and of the intact intergenic LINE1 (L1) that drives transcription of LCT14. At the bottom is an enlargement of the region including the LINE-1 (L1PA2; chr5:25,378,639–25,384,665) from which LCT14 originates with the regions (black bars) tested by bisulfite or hydroxymethylated DNA (hMeDIP) and chromatin (ChIP) immunoprecipitations and, below these, the LCT14 transcript (chr5: 25,384,485–25,384,958) and the region amplified for expression studies. All coordinates are from hg19 annotations; scale is in kb. (B) Expression of LCT14 measured by real-time RT-PCR and expressed relatively to the geometric mean of 3 reference genes in matched normal (dark gray) and tumor (light gray) tissues from 4 colorectal cancer patients (left panel) and of 5 colorectal cancer cell lines (right panel). (C) Methylation levels measured by bisulfite sequencing in the paired normal and tumor tissues of the 4 patients (left panel) and cell lines (right panel) described in B.

### The co-occurrence of DNA methylation and expression of LCT13 and LCT14 is not due to increased levels of hydroxymethylcytosine

**Figure 4.**
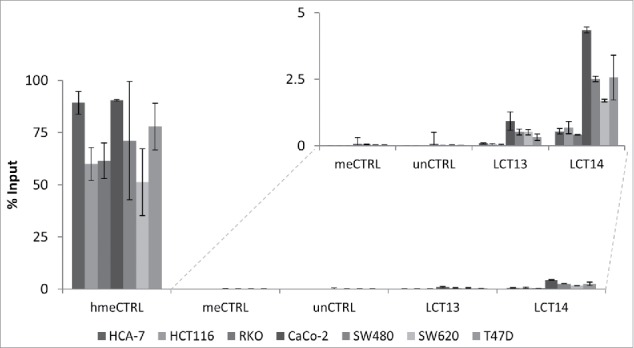
Analysis of 5hmC at LCT13 and LCT14 in cancer cell lines. Levels of hydroxymethylcytosine (hmeC) obtained by hMeDIP and expressed as % of Input. hmeCTRL: hydroxymethylated control DNA; meCTRL: methylated control DNA; unCTRL: unmethylated control DNA. The inset shows an enlargement of the region of the graph without the positive hmeCTRL indicating that some cell lines (Caco-2, SW480, SW620 and T47D) show minor enrichment at LCT13 and LCT14 L1ASPs, relative to the negative controls and the negative cell lines.

To address whether the observations made at LCT13 were unique to this particular locus, we analyzed the relationship between DNA methylation and expression of another intergenic LCT (LCT14), which we previously identified as initiating from the L1-ASP of an intergenic L1PA2 on chromosome 5 (Fig. 3A).^15^ We selected matched normal (N) and tumor (T) tissues from 4 CRC donors, 2 that did not show any LCT14 expression in N or T (148 and 218), one that expressed LCT14 in both N and T (153) and one that did not express LCT14 in N but did in T (205) (Fig. 3B, left panel). DNA methylation levels were above 80% in all tissue samples, including those expressing LCT14 (Fig. 3C left panel; Fig. S4A). We also compared LCT14 expression and methylation in 5 CRC and 4 BC cell lines. We could detect expression of LCT14 only in CaCo-2 and SW620 (Fig. 3B, right panel); however, all CRC lines showed >85% methylation at this LCT (Fig. 3C, right panel; Fig. S4A). In BC cells, we observed the predicted pattern of no expression and high methylation in HMEC, and high expression and no methylation in T47D; however, we saw expression of LCT14 in MCF7 cells that have levels of methylation comparable to those in HMEC, and some expression in HCC1954 with about 45% methylation (Fig. S4B and C). These observations are consistent with those made at LCT13 suggesting that at least in the context of CRC, transcription and methylation of LCT14 are not always mutually exclusive (Fig. S5). However, caution must be taken when interpreting these results because we used bisulfite sequencing to assess the levels of methylation. It has been shown that this method is not able to distinguish between methylated (5mC) and hydroxymethylated (5hmC) cytosines, an intermediate product of cytosine demethylation.^39^ To address this issue, we performed hydroxymethylated DNA immunoprecipitations (hMeDIP) on the 6 CRC cell lines and on T47D cells that are not methylated at either LCTs. When compared with the levels detected for the hydroxymethylated DNA control, the levels of 5hmC at the 2 LCTs are very low in all cell lines (Fig. 4), consistent with the reported loss in 5hmC in cultured cells.^40^ To better appreciate differences between the cell lines, we plotted the results removing the positive control and found that levels are very low, below 5% of Input, with slightly higher levels found at LCT14 than at LCT13 (Fig. 4, inset). Interestingly, the 3 MSS lines and T47D have higher 5hmC levels when compared with the MSI lines, though these differences do not reach statistical significance for LCT13. It is important to note that T47D that has very low to undetectable DNA methylation by bisulfite sequencing at LCT14 shows levels of 5hmC that are comparable to those seen at SW480 which do not express LCT14 and have high levels of DNA methylation. Similarly, SW480 has the highest levels of 5hmC at LCT13, but do not express this LCT. These data indicate that there is no 5hmC enrichment at the L1–5′UTR of LCT13 and LCT14 suggesting that in some cell lines these LCTs can be active and methylated.

**Figure 5.**
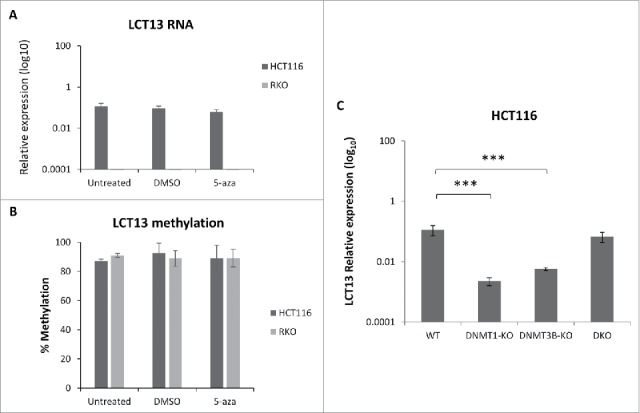
Effects of DNA methylation inhibition in colon cancer cell lines. Expression (A) and methylation (B) of LCT13 in HCT116 cells untreated or treated with DMSO vehicle alone or 1 μM 5-aza in DMSO. Treatment with 5-aza has no effect on LCT13 expression levels; no overall changes in the levels of DNA methylation are seen in either cell line. (C) Expression profile of LCT13 in HCT116 that are either wild type or lacking DNA methyltransferase 1 (DNMT1-KO), or 3B (DNMT3B-KO), or both DNMT1 and 3B (DKO). *P* values were calculated by one-way ANOVA. ***: *P < 0.001*

### Effects of DNA methylation inhibition on LCT13 expression

To further investigate the relationship between methylation and expression of LCT13 we focused on HCT116 and RKO cells, established cell lines that have been widely used to study DNA methylation using inhibitors.^41,42^ We treated these cells with the DNA methylation inhibitor 5-azacytidine (5-aza) and confirmed that the treatment did not affect expression of the 3 reference genes used for quantification of RT-PCR (Fig. S6A). Expression of *TFPI-2* upon 5-aza treatment, a gene known to be induced in cancer cells upon this treatment,^43^ confirmed its effectiveness (Fig. S6B). Comparison of LCT13 expression and methylation profiles in untreated, 5-aza or vehicle alone (DMSO) treated cells revealed that the 5-aza treatment has no effect on LCT13 expression (Fig. 5A) or levels of DNA methylation (Fig. 5B; Fig. S6C) in either cell lines.

**Figure 6.**
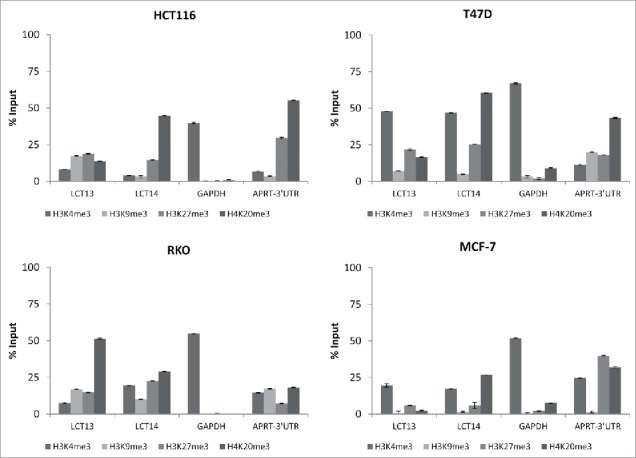
Histone modifications at LCT13 and LCT14 in cancer cell lines. ChIP assays performed using antibodies against active (H3K4me3) and repressive (H3K9me3, H3K27me3, H4K20me3) histone marks in LCT13 positive and LCT14 negative HCT116 cells (top left panel), in LCT13 and LCT14 negative RKO cells (bottom left panel), and in LCT13 and LCT14 positive T47D (top right panel) and MCF-7 (bottom right panel) cells. GAPDH is a promoter Taqman assay used as a positive control for H3K4me3 and APRT-3′UTR has been previously shown to be enriched at repressive mark H4K20me3.

The apparent lack of direct relationship between DNA methylation and regulation of LCT13 was further supported by the analysis of LCT13 expression in HCT116 lacking the maintenance (DNMT1) or *de novo* (DNMT3B) or both DNA methyltransferases.^44,45^ Surprisingly, a statistically significant decrease in the levels of LCT13 is seen in HCT116 cells lacking either DNMT1 or DNMT3B relatively to wild type cells while no significant differences are seen in HCT116 lacking both DNMTs (Fig. 5C), consistent with the data from 5-aza treatment. Taken together these data suggest that in HCT116 cells regulation of the promoter of LCT13 relies on a more complex regulatory mechanism including factors other than DNA methylation.

### Profile of histone modification at LCT13 and LCT14 in cancer cells

To gain a deeper insight into the epigenetic regulation of LCT13 and LCT14, we performed chromatin immunoprecipitation (ChIP) assays using antibodies raised against the active chromatin histone modification H3K4me3, and the heterochromatin associated marks H3K9me3, H3K27me3, and H4K20me3. We tested CRC cell lines HCT116 that express LCT13 but not LCT14, and RKO that are negative for both LCTs (Figs 1 and 3). As a comparison, we also analyzed the BC cell lines T47D and MCF-7, that express both LCTs but show no or some methylation at the 2 L1–5′UTR, respectively (Figs. S2 and S4). The modification showing a more evident difference between expressed and non-expressed LCTs in the CRC lines is H4K20me3. LCTs that are not expressed (LCT14 in HCT116 and LCT13 and 14 in RKO) have an enrichment in H4K20me3 that is >25% Input (Fig. 6, top and bottom left panels), a finding consistent with this modification being enriched at LINE-1 sequences.^46^ Surprisingly we found that enrichment in H3K4me3 at LCT13 in HCT116 cells was very modest, despite the fact that this LCT is expressed in these cells (Fig. 1B); however, this finding is in agreement with the high levels of DNA methylation at LCT13 in HCT116 cells (Fig. 1C) and with the reported mutual exclusivity of these 2 modifications.^47,48^ BC cells T47D that are not methylated at either loci, show the highest levels of H3K4me3 at both L1–5′UTRs (Fig. 6, top right panel), while lower levels are seen in MCF-7 cells (Fig. 6, bottom right panel). Levels of H4K20me3 at the LCT13 5′UTR are lower than those of H3K4me3 in both T47D and MCF-7 cells; by contrast, levels of H4K20me3 at the LCT14 5′UTR in both cell lines are comparable to those of H3K4me3 (Fig. 6, top and bottom right panels). Interestingly, LCT13 that is not upregulated by 5-aza in RKO cells (Fig. 4A) has low H3K4me3 and high H4K20me3 similar to what observed at the silenced LCT14 in HCT116 cells (Fig. 6, top and bottom left panels). Instead, LCT14 that is expressed upon 5-aza treatment in RKO (Fig. S7A), displays comparable H3K4me3 and H4K20me3 levels of enrichment in these cells (Fig. 6, bottom left panel), a pattern similar to that observed at the expressed LCT13 in HCT116 and at the expressed LCT14 in T47D and MCF-7 cells (Fig. 6). Although no overall changes in methylation levels are observed between treated and untreated cells (Fig. S7B), LCT14 expression in 5-aza treated RKO cells is associated with decrease in methylation at a specific CpG site (Fig. S7C). This data suggest that that the relative enrichment in H4K20me3 at LCTs is better than DNA methylation at predicting whether a LINE-1 promoter is active or likely to be sensitive to 5-aza treatment.

## Discussion

Cancer is a complex genetic disease, resulting from the accumulation of several genetic mutations and epigenetic changes that allow cells to overcome the normal biologic hurdles and defense mechanisms limiting growth and division. Decreases in methylation at repetitive DNA is probably the most accepted paradigm among the global changes in cancer, so much so that loss of LINE-1 methylation is repeatedly used as a surrogate biomarker for measuring global DNA hypomethylation.^49–52^ Nevertheless, a significant decrease in LINE-1 methylation has not been seen in all cancer types. Susceptibility to LINE-1 hypomethylation appears to be cell and tissue specific and might be dependent on cancer type or subsets of a specific cancer type.^53–55^ Moreover, emerging evidence suggest that not all LINE-1s are regulated in the same way.^56^ Global LINE-1 methylation studies are unable to provide evidence for a relationship between methylation at individual LINE-1 and their activity. The observation that genes commonly downregulated in cancer are more likely to contain an intragenic LINE-1 indicates that the positioning of the LINE-1 is an important factor in its regulation.^57^ In agreement with genome-wide studies, loss of methylation at intronic LINE-1 situated within *c-MET, RAB3IP*, and *CHRM3* genes was found to lead to activation of transcription from L1-ASP.^36,37,58^ However, recent reports have shown that in mouse adult, embryonic and primordial germ cells loss of LINE-1 methylation does not always coincide with activation of transcription.^34,35,59^ In addition, the repressive role of DNA methylation at LINE-1 promoters was questioned in a study in early development in mouse embryos that identified loss of the active histone H3K4me3 mark as the reason for decrease in L1 expression at the 8-cell stage compared with the 2-cell stage.^60^ Although these observations were made in mouse, which have LINE-1s that are different from humans, the majority of the studies in human colorectal cancer also point to the lack of a direct linear relationship between LINE-1 hypomethylation levels and tumor stage,^27,61–63^ suggesting that, in man and in mouse, DNA methylation cannot be the key regulator of expression from all LINE-1s.

In this study, methylation levels at the L1–5′UTR of 2 intergenic LINE-1s were investigated and compared with the levels of their specific LCT transcripts, LCT13 and LCT14. It must be noted that we were able to analyze region 1–436 of the L1–5′UTR of the 2 LCTs and, therefore, we did not include the L1-ASP promoter core activity that has been proposed to map between positions 450 and 600 of the 5′UTR.^13,14^ A direct correlation was not observed between LCT13 or LCT14 expression and methylation in the CRC patient samples, where, except for 2 individuals, LCT13 and LCT14 methylation levels were found to be high in both tumors and matched normal tissues regardless of their expression (Figs 1 and 3). The lack of a direct relationship between DNA methylation and promoter activity within the L1–5′UTRs of LCT13 and LCT14 was also observed in the breast and colon cancer cell lines analyzed, albeit in breast cells there was evidence of an inverse correlation between methylation and expression levels. Differential methylation and expression levels of LINE-1 elements in different cell lines and during different stages of development have been documented previously.^64–66^ However, it is also possible that changes in LINE-1 methylation levels can have different effects on an individual LINE-1 promoter activity depending not only on its unique location and surrounding DNA but also on the cell type and whether the transcription machinery or silencing complexes are available. We have further characterized LCT13 by 5′RACE in these cell lines and we found that LCT13 transcription starts within L1–5′UTR in all expressing cell lines, and DNA methylation alone does not seem to be sufficient to prevent transcription (Fig. 2). Consistent with a lack of relationship between methylation and expression of LCT13, 5-aza treatment did not appear to affect L1-ASP methylation levels nor LCT13 expression in HCT116 or RKO cells; however, caution must be taken when interpreting these data given that at the dose used there was no reduction of methylation at the LCT loci tested. Nevertheless, in agreement with the 5-aza findings, we found that HCT116 cells lacking both DNMT1 and DNMT3B show no changes in LCT13 expression relative to wild type cells; however, knockout of either DNMTs led to a significant decrease in LCT13 expression (Fig. 5).

Histone modification profiling at the LCT13 and LCT14 L1–5′UTRs (Fig. 6) suggest H4K20me3 as a better indicator than DNA methylation to determine LCT expression or sensitivity to 5-aza. This modification has been previously shown to be enriched at LINE-1s in CRC,^67^ and a recent study has reported variation in the levels of H4K20me3 at different LINE-1 subtypes.^68^ We found that in HCT116 and RKO cells we could not induce expression of the LCTs that had a relative enrichment in H4K20me3 at levels above 40% of Input (LCT14 and LCT13, respectively) with 5-aza under the conditions used (Fig. 5 and Fig. S7). Future experiments will be necessary to demonstrate the correlation between H4K20me3 and LCT activation, for example, by testing if loss of H4K20me3 (SUV4–20 knockdown) in cells in which LCTs are repressed is sufficient to upregulate expression. Moreover, extending the repertoire of unique LINE-1s analyzed will be necessary to confirm the generality of this finding. This knowledge will be very important in the context of clinical applications of therapeutic agents such as 5-aza. For example, a therapy likely to promote activation of the LINE-1 promoter driving LCT13 or that intragenic to *cMET* could increase the risk of developing cancer by promoting silencing of the metastasis suppressor gene *TFPI-2* or expression of the oncogenic isoform L1-MET.^37,38^

In conclusion, our data indicate that DNA methylation may not always play a key role in silencing the L1–5′UTRs of intergenic LINE-1s similar to those driving LCT13 and LCT14. It is more likely that the regulation of these promoters is achieved by a dynamic network and interplay between several factors that are compromised in some but not all cases of breast and colorectal tumors. Further investigations are needed to establish the other regulatory factors involved in LINE-1 promoter control and the exact role of DNA methylation in this process.

## Materials and methods

### Patient details and ethics

Ethical approval for this study was granted by the Derbyshire Research Ethics Committee for the collection of normal and cancer colorectal tissues from patients who underwent surgical resection at the Royal Derby Hospital (Derby, UK) after informed written consent was obtained. All tumors collected were large specimens of size ranging from 2.25 cm^3^ to 42 cm^3^; the sections used had ≥ 75% tumor cells

### Cell lines cultures and 5-azacytidine treatment

Cancer cell lines were obtained from ECACC (HCA-7, HCT116, CaCo2, SW620, SW480, MCF-7) or ATCC (RKO, HCC1954) and cultured according to the guidelines from the providers. HMEC (Invitrogen) were kindly provided by Dr. Allegrucci as frozen cell pellets ready for nucleic acid extraction. DNA samples from the cell lines were sent to Bio-Synthesis, Inc. for STR profiling to confirm cell line identity.

HCT116 and RKO Cells were treated for 72 h with 1 µM 5-azacytidine (Sigma, Cat. no. A2385) replacing the media every 24 h with fresh media containing 1 µM 5-aza. The HCT116 DNMT knockout cells were kindly donated by B. Vogelstein for the work published in^69^ and their identity confirmed at that time via expression analysis and DNA methylation profiles. Cell pellets used for the analyses described in the present manuscript were prepared by Dr. Ottaviano from the same cultures, subsequently checked by expression analysis and DNA methylation profiles (manuscript in preparation).

### Nucleic acids extractions and RT-PCR

DNA and total RNA was extracted from tissues and cell lines using Allprep RNA/DNA mini kit (QIAGEN, Cat. no. 80204) according to the manufacturer's instructions, including the on column DNaseI treatment (QIAGEN, Cat. no. 79254) for RNA extraction. RNA integrity was evaluated by agarose gel electrophoresis. Reverse transcription was performed on 60 ng of RNA with random primers using High Capacity cDNA Reverse Transcription Kit (Applied Biosystems, Cat. no. 4374966) in a final volume of 20 μl and following manufacturer's instructions. An aliquot (2 μl) of the reverse transcription reaction was used for real-time PCR and each sample was analyzed in triplicate. Real time PCR was performed using custom designed Taqman assays for the LCTs and commercially available Taqman assays for the house keeping genes (Applied Biosystems). The thermocycling parameters were as follows: 10 min at 95°C, followed by 45 cycles of 15 s at 95°C, and 1 min at 60°C. Details of all Taqman assays used can be found in Table S1. Processing and analysis of real-time PCR data was performed on GenEx software (bioMCC, Germany). Levels of LCT RNA expression were expressed relatively to the geometric mean of 3 reference genes (*HPRT, PGK*, and *GAPDH*; see Supplementary Methods and Fig. S1).

### Bisulfite

Bisulfite conversion of DNA was performed using EZ DNA Methylation-Gold Kit (Zymo research, Cat. no. D5005) according to the manufacturer's protocol. Primary and secondary PCRs were performed using ZymoTaq (Zymo Research, Cat. no. E2001) in a final volume of 25 µl, under the following conditions: 10 min incubation at 95°C before 40 cycles of 30 sec at 95°C, 1 min at 48°C (49°C for TFPI2 primary primer pair) and 1 min at 72°C. Finally, the samples were incubated at 72°C for 10 min. Bisulfite treated DNA (40 ng) was used in the primary PCR reaction; 1 µl of the primary PCR was used as template for the secondary PCR. All primers were used at a final concentration of 0.6 µM (see Table S2 for details of primers). PCR products were purified from the gel using QIAquick Gel Extraction kit (QIAGEN, Cat. no. 28704) and cloned using pGEM®-T Easy Vector System I (Promega, Cat. no. A1360). Transformation was performed using XL10 Gold ultra-competent cells (Agilent Technologies, Cat. no. 200315) followed by selection on ampicillin (Sigma-Aldrich, Cat. no. A9518) containing plates following standard protocols. Plasmid purification was performed using QIAprep Spin miniprep kit (QIAGEN, Cat. no. 27106) or PureYield™ Plasmid Miniprep System (Promega, Cat. no. A1223). Plasmid DNA was sent to Source Biosciences for Sanger sequencing. Sequencing data was analyzed using MacVector™ 8.0 and bisulfite analysis was performed using the BIQ analyzer excluding clones with estimated conversion rates below 90% and clones 100% identical to another clone unless the overall methylation state was close to 0% or 100%.^70^

### 5′RACE

5′RACE was performed as described previously,^15^ except that the LCT13 reverse transcription primer (NV009: 5′TTTGTCCTTTTCTGTCAGGTCCTC3′) and the gene specific primers for primary (NV010: 5′GCATCTTTTTGCCTGTTGTGGA3′) and secondary (NV011: 5′ATCTTTTTGCCTGTTGTGGAGG3′) PCR amplification were designed within exon 2 (Fig. 1A, bottom diagram). *APRT* was used as a positive control using the previously published primers.^15^ PCR products were gel purified, cloned, and sequenced as described above.

### hMeDIP

Immunoprecipitation of hydroxymethylated DNA was performed with a mouse monoclonal antibody against 5-hydroxymethylcytosine (5hmC) using the hMeDIP kit (Diagenode, Cat. no. C02010031) following the manufacturer's instructions. DNA samples were sheared at 4°C using a Diagenode Bioruptor. DNA (1 μg) was used for each IP; recovered DNA was analyzed by real-time PCR (Table S3). Hydroxymethylated, methylated, and unmethylated DNA controls and primers for their amplification were provided with the kit.

### Chromatin IPs

Chromatin Immunoprecipitation (ChIP) assays were performed using EZ-Magna ChIP™ A kit (Millipore, Cat. no. 17–408) according to the manufacturer's instructions. Antibodies raised in rabbit against H3K4Me3, H3K9me3, H3K27me3, and H4K20me3 were purchased from Diagenode (Cat. no. pAb-003–050, pAb-056–050, pAb-069–050 and pAb-057–050, respectively). Rabbit IgG was provided with the ChIP kit. Cells were crosslinked in their culture vessel with the appropriate media containing 1% formaldehyde (Sigma, Cat. no. F8775) for 10 min at room temperature. Cells were sonicated at 4°C using a Diagenode Bioruptor. Cells (10^6^) were used for each IP and the recovered material was analyzed by real-time PCR using custom designed Taqman assays for LCT13, LCT14, and APRT (Table S3). *GAPDH* primers were provided in the kit.

## Financial disclosure

Research was supported by the Derby Teaching Hospitals NHS Foundation Trust Colorectal Cancer Charitable Fund; and by Genetic Society summer scholarships to LMM and CP. NV-I was supported by a University Research Scholarship Doctoral Training Award from the University of Nottingham. Research in RRM laboratory is supported by the MRC. The funders had no role in study design, data collection and analysis, decision to publish, or preparation of the manuscript.

## Supplementary Material

KEPI_A_1300729_s02.pdf
