# Supplementary material for: Decoupling of DNA methylation and activity of intergenic LINE-1 promoters in colorectal cancer
Source: Epigenetics. 2017 Mar 16;12(6):465–75. doi: 10.1080/15592294.2017.1300729 (PMC5501206; doi:10.1080/15592294.2017.1300729)
Supplement: KEPI_A_1300729_s02.pdf [file kepi-12-06-1300729-s001.pdf]

Supplementary Information

Table of Contents

Supplementary methods 2

Selection of reference genes for expression analysis in colorectal cancer 2

Supplementary Figures 4

Supplementary Figure 1 4

Supplementary Figure 2 5

Supplementary Figure 3 6

Supplementary Figure 4 7

Supplementary Figure 5 8

Supplementary Figure 6 9

Supplementary Figure 7 10

Supplementary Tables 11

Supplementary Table 1- Primers used for real time PCR 11

Supplementary Table 2- Primers used for bisulphite sequencing assays 11

Supplementary Table 3- Primers used for PCR of IPs 12

Supplementary methods

Selection of reference genes for expression analysis in colorectal cancer

A necessary initial step was to develop a method for accurately measure the levels of expression of the chosen LCTs in the matched normal and tumour mucosae from CRC patients. A common method for quantifying real time PCR data is to measure expression of the RNA of interest relatively to that of a housekeeping gene used as endogenous control under the assumption that expression of the housekeeping gene (also known as reference gene) is the same in the different samples tested. However, it has become evident that reference genes vary in expression levels in different tissues and disease states.[1] Recent reports have shown that this problem can be overcome by identifying for each experimental set up a set of three reference genes whose geometric mean does not change between the samples to be analysed. The geometric mean of the chosen reference genes can then be used for normalisation thus enabling the comparison of RNA expression levels between tissue samples from different individuals and disease states.[2, 3] Two previous studies have investigated the best reference genes to be used for expression studies in CRC samples with different outcomes,[4, 5] highlighting the importance of validating the reference genes in individual experimental set-ups.

In order to choose the three best reference genes, six candidate reference genes (*PGK*, *HPRT*, *GAPDH*, *RPLO*, *IPO8* and *PPIA*) were chosen amongst those reported as more stable in CRC.[4, 5] Reverse transcription followed by real time PCR using commercially available Taqman assays (Supplementary Table 1) was performed in triplicate on total RNA extracted from tumour and matched normal mucosa from 10 CRC patients and expression stability of these genes was analysed using GeNorm and NormFinder.[2, 6] GeNorm performs pairwise comparisons between the housekeeping genes of the variation observed for each reference gene amongst the samples tested and provides an M value, with lower M values indicating higher expression stability. This software indicated *PGK1* and *GAPDH* as the top two most stable reference genes across the cohort as well as between normal and tumour samples and *HPRT* the next most stable reference gene (Supplementary Figure 1A). NormFinder measures variation of expression of each gene relatively to the average of expression of all genes in all samples and produces a standard deviation (SD) value for each gene, with the gene with the lowest SD as the best reference gene. In addition NormFinder can also calculate the accumulated SD expected if multiple reference genes were used and thus provide a tool to determine the minimum number of genes required to reduce the effects of random variations amongst the genes' expression. NormFinder indicated *PGK1* as the best reference gene for our cohort with *GAPDH* and *HPRT* as second and third best genes (Supplementary Figure 1B, left panel). It also indicated that using *PGK1* and *GAPDH* would be sufficient for normalisation (Supplementary Figure 1B, right

panel), however *HPRT* was also used in this study to conform to the MIQE (Minimum Information for Publication of Quantitative Real-Time PCR Experiments) guidelines suggesting the use of a minimum of 3 reference genes in expression studies.[7]

Supplementary References

1. Hruz T, Wyss M, Docquier M, Pfaffl MW, Masanetz S, Borghi L, Verbrugghe P, Kalaydjieva L, Bleuler S, Laule O, et al. RefGenes: identification of reliable and condition specific reference genes for RT-qPCR data normalization. BMC genomics 2011; 12:156.
2. Vandesompele J, De Preter K, Pattyn F, Poppe B, Van Roy N, De Paepe A, Speleman F. Accurate normalization of real-time quantitative RT-PCR data by geometric averaging of multiple internal control genes. Genome biology 2002; 3:RESEARCH0034.
3. Jacob F, Guertler R, Naim S, Nixdorf S, Fedier A, Hacker NF, Heinzelmann-Schwarz V. Careful selection of reference genes is required for reliable performance of RT-qPCR in human normal and cancer cell lines. PLoS One 2013; 8:e59180.
4. Sorby LA, Andersen SN, Bukholm IR, Jacobsen MB. Evaluation of suitable reference genes for normalization of real-time reverse transcription PCR analysis in colon cancer. J Exp Clin Cancer Res 2010; 29:144.
5. Rubie C, Kempf K, Hans J, Su T, Tilton B, Georg T, Brittner B, Ludwig B, Schilling M. Housekeeping gene variability in normal and cancerous colorectal, pancreatic, esophageal, gastric and hepatic tissues. Mol Cell Probes 2005; 19:101-9.
6. Andersen CL, Jensen JL, Orntoft TF. Normalization of real-time quantitative reverse transcription-PCR data: a model-based variance estimation approach to identify genes suited for normalization, applied to bladder and colon cancer data sets. Cancer research 2004; 64:5245-50.
7. Bustin SA, Benes V, Garson JA, Hellemans J, Huggett J, Kubista M, Mueller R, Nolan T, Pfaffl MW, Shipley GL, et al. The MIQE guidelines: minimum information for publication of quantitative real-time PCR experiments. Clin Chem 2009; 55:611-22.

Supplementary Figures

Supplementary Figure 1

A

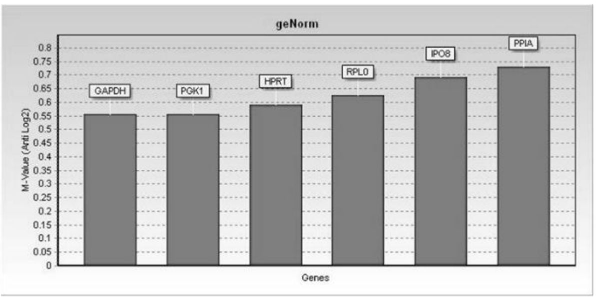

B

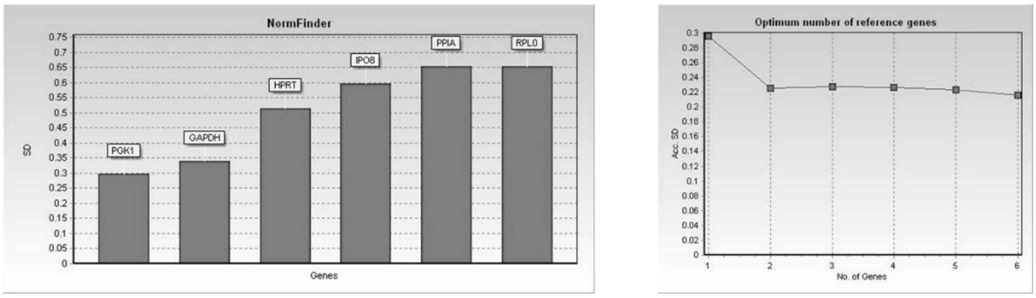

Supplementary Figure 1 – Expression of six reference genes was tested in tumour and matched normal tissue of ten CRC patients to select the most appropriate for quantitative analysis. (**A)** geNorm software output showing that GAPDH and PGK1 have the most stable expression within the CRC samples closely followed by HPRT. (**B)** NormFinder software plot demonstrating PGK1 as the most stable expression amongst all the samples closely followed by GAPDH (left side) and two genes are sufficient for normalization (right side).

Supplementary Figure 2

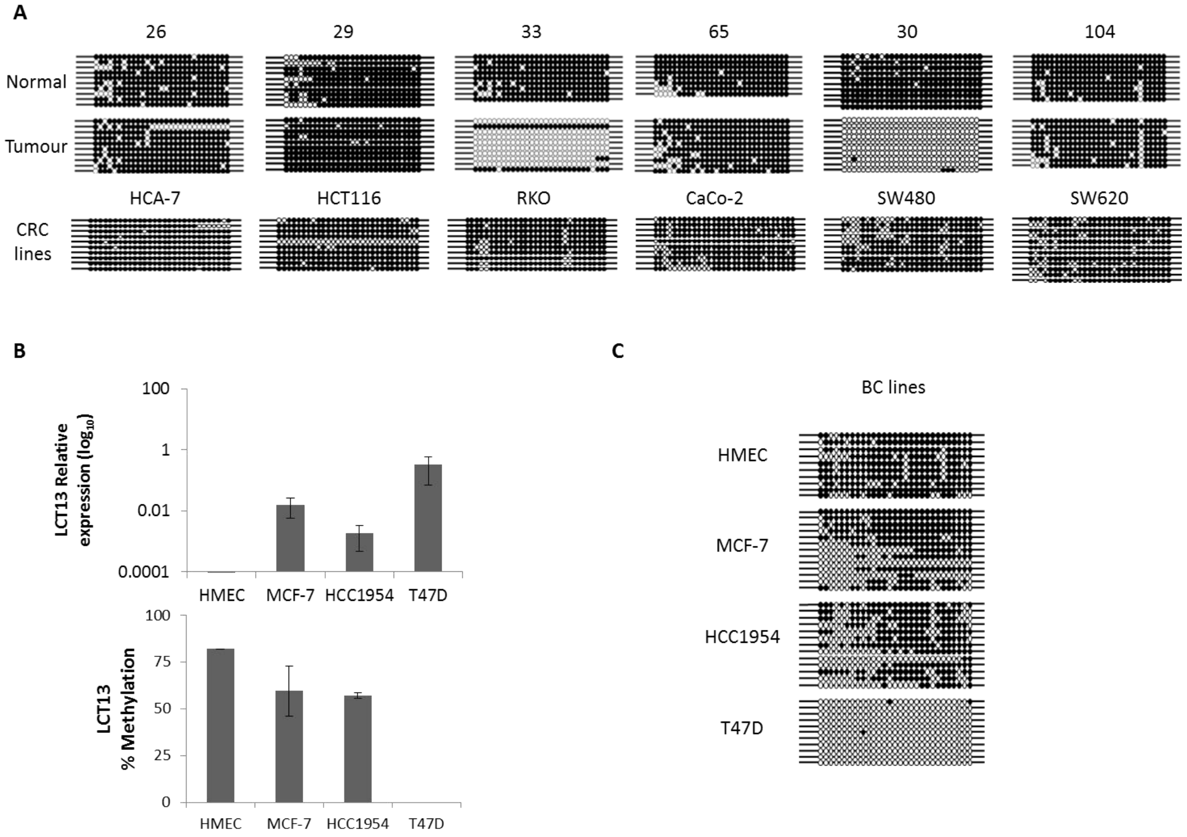

Supplementary Figure 2 – (**A**) Methylation data from bisulfite sequencing of a 495bp amplicon within LCT13 L1ASP including 29 CpGs ('bisulfite' in Figure 1A bottom diagram and Figure 2A) performed on matched normal and tumour tissue of the six colorectal cancer patients and cell lines described in Figure 1; individual clones from one representative experiments for each sample are shown. (**B**) Bar charts showing expression (top panel) and methylation (bottom panel) of LCT13 in 3 breast cancer cell lines (MCF-7, HCC1954, T47D) and in human mammary epithelial cells (HMEC) used as normal control. (**C**) Methylation data from bisulfite sequencing of the region described in (A) in the panel of breast cancer cell lines; individual clones from one representative experiments for each sample are shown.

Supplementary Figure 3

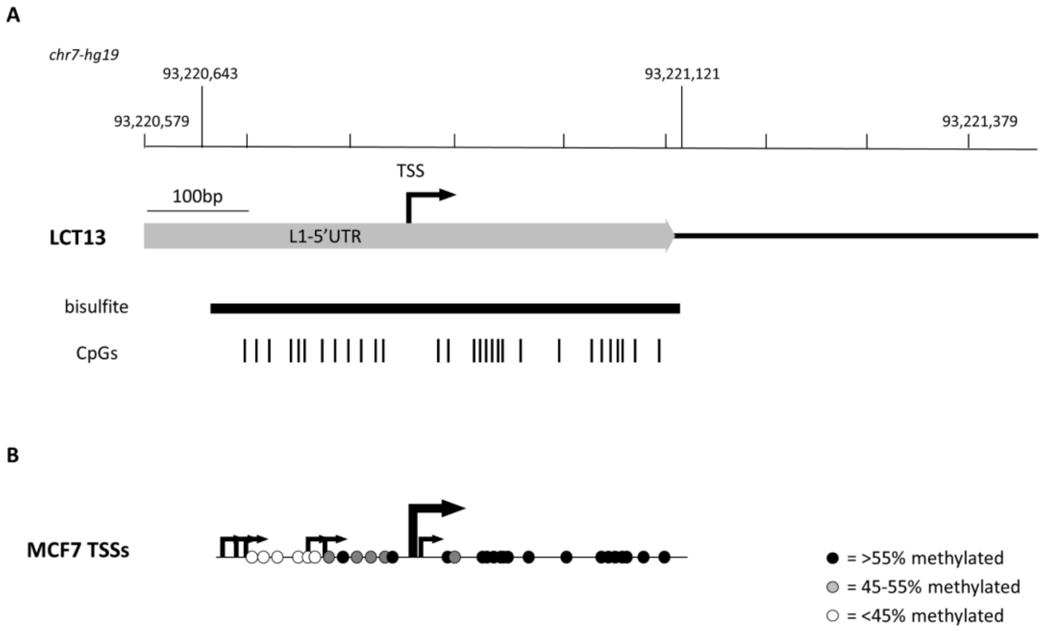

Supplementary Figure 3 – (**A**) Schematic diagram showing the 5'UTR of the L1PA2 driving LCT13 (chr7: 93,220,579-93,221,079) with indicated the regions analysed by bisulfite sequencing (black bar; chr 7: 93,220,643-93,221,121) and the positions of the 29 CpG sites (vertical black lines) within it. All coordinates are from hg19 annotations and the scale is in base pairs (bp). (**B**) Diagram combining the average of methylation at each CpG with the location of TSSs (bent arrows) found by 5'RACE in MCF7 cells. The thick, tall bent arrow represent the major TSS; additional weaker TSS can be identified as indicated by the smaller bent arrows, demonstrating the presence of scattered initiation of transcription from the L1ASP promoter.

Supplementary Figure 4

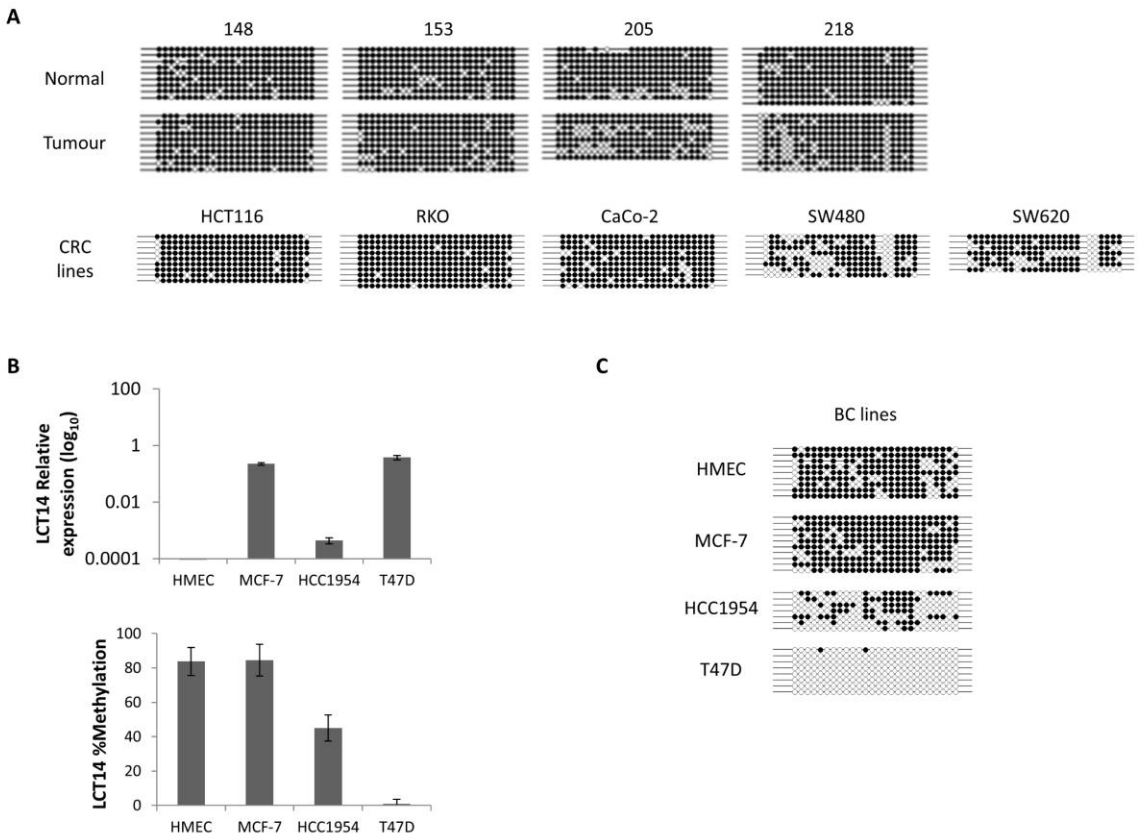

Supplementary Figure 4 – (**A**) Methylation data from bisulfite sequencing of a 459bp amplicon within LCT14 L1-5'UTR including 26 CpGs performed on matched normal and tumour tissue of the four colorectal cancer patients and the colon (CRC lines) and breast (BC lines) cell lines described in Figure 3; individual clones from one representative experiments for each sample are shown. (**B**) Top panel: Expression of LCT14 measured by real time RT-PCR and expressed relatively to the geometric mean of three reference genes in four breast cell lines. Bottom panel: Methylation levels measured by bisulfite sequencing in the breast cancer cell lines described in A. (**C**) Methylation data from bisulfite sequencing of the region described in (A) in the panel of breast cancer cell lines; individual clones from one representative experiments for each sample are shown. Black circles = methylated CpG, empty circles = unmethylated CpG.

Supplementary Figure 5

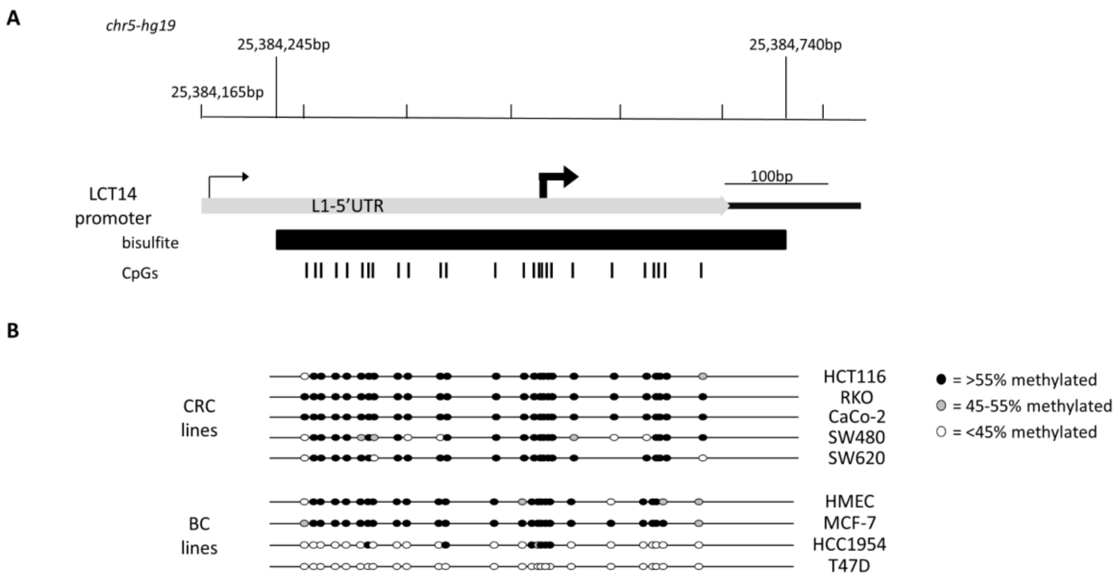

Supplementary Figure 5 – (**A**) Schematic diagram showing the first 500 nucleotides of the 5'UTR of the L1PA2 driving LCT14 with indicated the two transcription start sites for LCT14 described in Cruickshanks and Tufarelli, 2009 (thick bent arrow= strong site; thin bent arrow=weak site). Above the drawing is the scale in bp with annotated the corresponding co-ordinates on chr5 in hg19. 'bisulfite' is the 495bp region analysed by bisulfite sequencing (black bar) and 'CpGs' the positions of the 26 CpG sites (vertical black lines) within it. (**B**) Lollipops diagrams summarising the average methylation at each of the 26 CpG site analysed in five colorectal (CRC) and four breast (BC) cancer cell lines.

Supplementary Figure 6

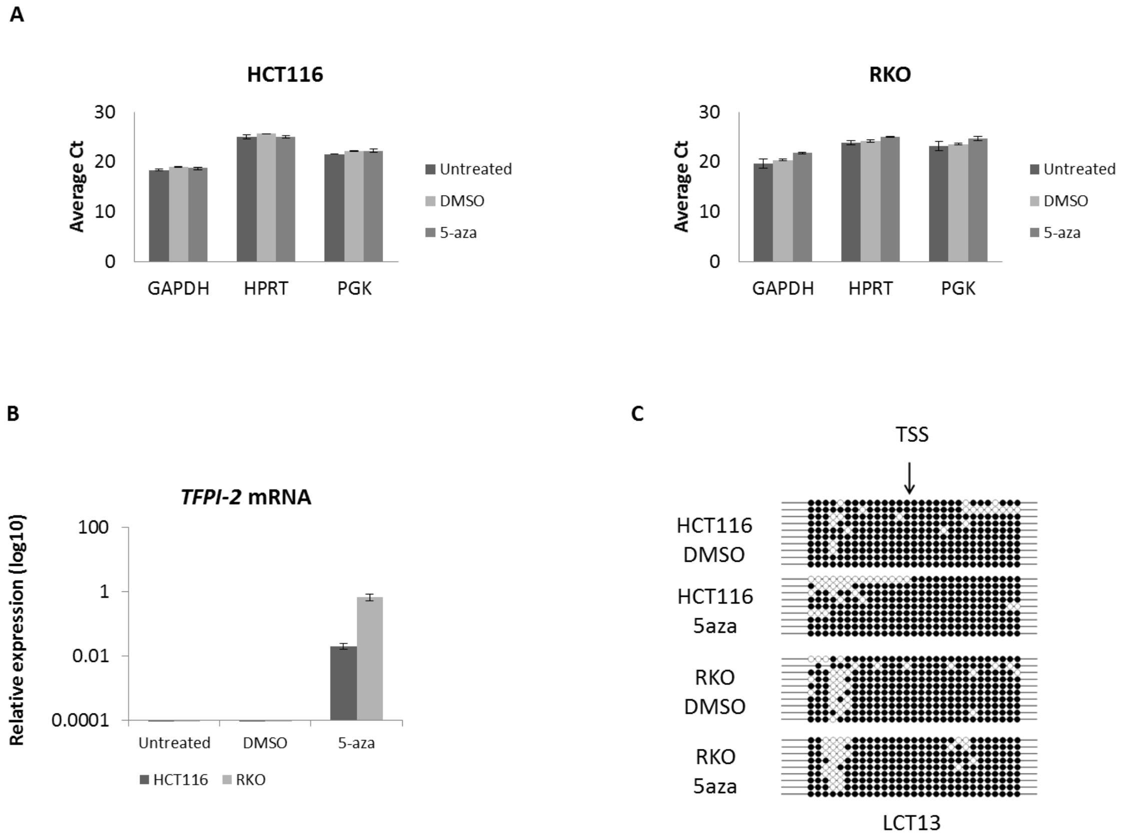

Supplementary Figure 6 – (**A**) 5-aza treatment of HCT116 (left panel) and RKO (right panel) cells does not affect expression of the 3 reference genes (GAPDH, HPRT and PGK) used for quantification of real time RT-PCR data. (**B**) 5-aza treatment leads to increased expression of *TFPI-2* consistent with 5-aza treatment having been successful as expression of this gene has been previously shown to be induced by 5-aza treatment in a variety of cell lines. (**C**) Methylation data from bisulfite sequencing of LCT13 in DMSO and 5-aza treated HCT116 and RKO cells; individual clones from one representative experiments for each sample are shown.

Supplementary Figure 7

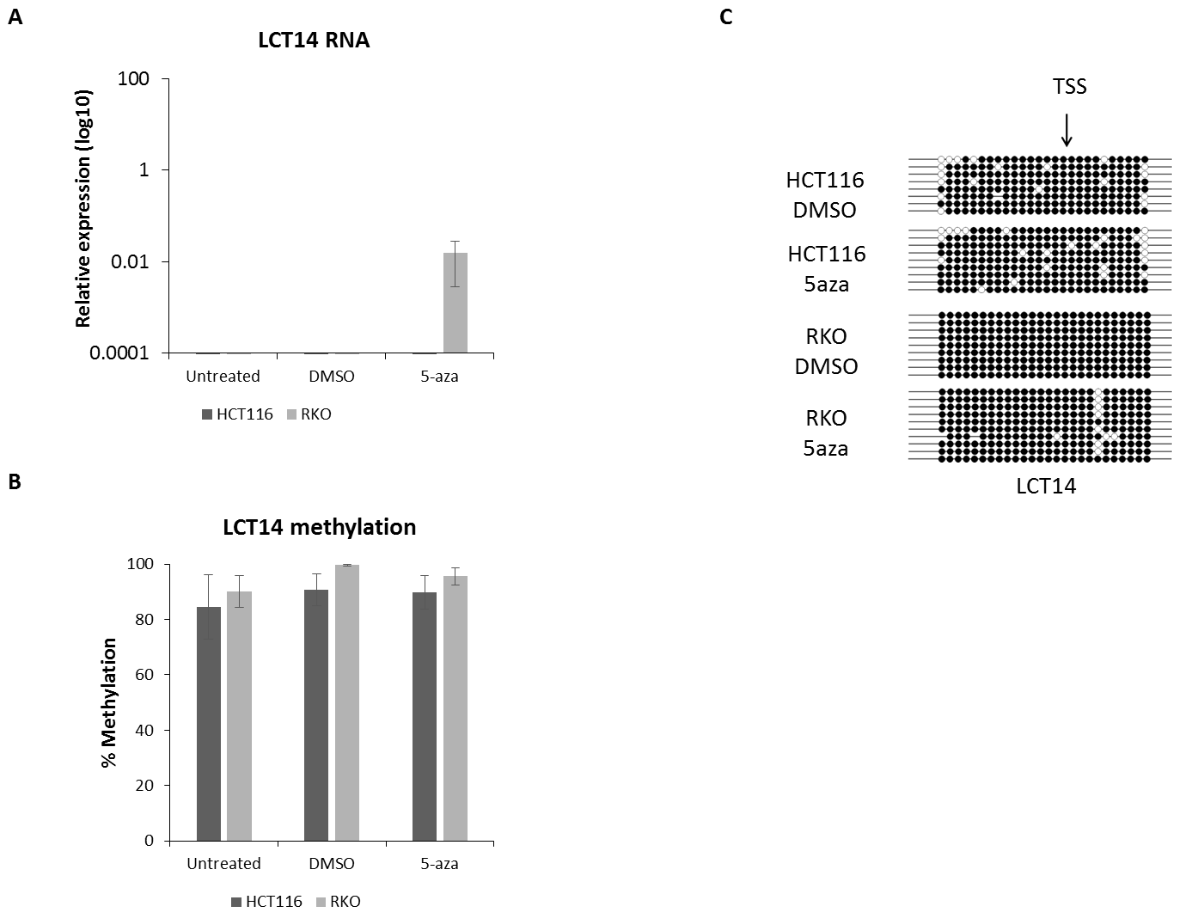

Supplementary Figure 7 – Expression (**A**) and methylation (**B**) of LCT14 in HCT116 cells untreated or treated with DMSO vehicle alone or 1uM 5-aza in DMSO. (**C**) Methylation data from bisulphite sequencing of LCT14 in DMSO and 5-aza treated HCT116 and RKO cells; individual clones from one representative experiments for each sample are shown.

Supplementary Tables

Supplementary Table 1- Primers used for real time PCR

Assay	ID no. / Sequence 5'-3' [a]	Slope	R2	Efficiency (%)
GAPDH	Hs02258991_g1 (ABI)	-3.446	0.9963	95
PGK1	Hs00943178_g1 (ABI)	-3.562	0.998	91
HPRT	Hs01003267_m1 (ABI)	-3.315	0.9992	100
PPIA	Hs04194521_s1 (ABI)	-3.413	0.9983	95
RPLP0	Hs04189669_g1 (ABI)	-3.287	0.9949	102
IPO8	Hs00183533_m1 (ABI)	-3.595	0.9949	90
TFPI-2	Hs00197918_m1 (ABI)	-3.573	0.9904	94
LCT13	GCTCCTATTCGGCCATCTTG<br>TCCTTTTCTGTCAGGTCCTCAATA<br>probe: CTCCACAACAGGCAAA	-3.258	0.9948	103
LCT14	CCTCTACTTCTGGAATCCATTGAGA<br>TGGTCAGAGGCACACAAGAAAG<br>probe: CATGCAACCATGTTCA	-4.460	0.9865	68

Note: (a) all Taqman probes used are conjugated with FAM

Supplementary Table 2- Primers used for bisulphite sequencing assays

LCT	PCR type	Primer	Sequence 5' – 3'	Product length	CpGs
**LCT13**	Primary	Hc072a	AGTTAAATTATTGATTTTGAGAGG	523bp	29
		Hc072b	CCTTACAATTTAATCTCAAACTAC		
	Nested	Hc072c	GATTTTGAGAGGTTAAGGAAGATAG	495bp	29
		Hc072d	CAAACTACTATACTAACAATCAACC		
**LCT14**	Primary	Hc073a	TATTTTAGAAATTGGGAGAGG	511bp	26
		Hc072b	CCTTACAATTTAATCTCAAACTAC		
	Nested	Hc073b	TAAAATAAGTAGGGAGAAAAAGAG	459bp	26
		Hc073c	CTCAAACTACTATACTAACAATCAAC		

Supplementary Table 3- Primers used for PCR of IPs

Assay	Sequence 5′ – 3′
**LCT13-IP**	GCCTTCTTGCATTGGATTCTTC
	GGCCCAATCCACGAGAGA
	probe: CATAAACCAGACTGCTCA
***APRT*-IP**	GCCTCCCAGCCCAACATC
	GCAGTTGCCCAAGGCTGATA
	probe: CAGCTGGATCCCAGGGA
**LCT14**	see Supplementary Table 1
GAPDH	Provided with EZ-Magna ChIP A Kit
**hme-ctrl**	Provided with hMeDIP Kit
**me-ctrl**	Provided with hMeDIP Kit
**un-ctrl**	Provided with hMeDIP Kit
